# Complex Elbow Dislocations and the “Terrible Triad” Injury

**DOI:** 10.2174/1874325001711011394

**Published:** 2017-11-30

**Authors:** Alistair D.R. Jones, Robert W. Jordan

**Affiliations:** 1 Department of Trauma and Orthopaedics, Worcestershire Royal Hospital, Charles Hastings Way, WR5, Worcester, 1DD, UK; 2 Coventry and Warwickshire Shoulder and Elbow Unit, University Hospitals Coventry & Warwickshire NHS Trust, Clifford Bridge Road, Coventry, CV2 2DX, UK

**Keywords:** Elbow, Complex dislocation, Terrible triad, Ulno-humeral joint, Elbow dislocations

## Abstract

**Background:**

The elbow is the second most commonly dislocated joint in adults and up to 20% of dislocations are associated with a fracture. These injuries can be categorised into groups according to their mechanism and the structures injured.

**Methods:**

This review includes a literature search of the current evidence and personal experiences of the authors in managing these injuries.

**Results:**

All injuries are initially managed with closed reduction of the ulno-humeral joint and splinting before clinical examination and radiological evaluation. Dislocations with radial head fractures should be treated by restoring stability, with treatment choice depending on the type and size of radial head fracture. Terrible triad injuries necessitate operative treatment in almost all cases. Traditionally the LCL, MCL, coronoid and radial head were reconstructed, but there is recent evidence to support repairing of the coronoid and MCL only if the elbow is unstable after reconstruction of lateral structures. Surgical treatment of terrible triad injuries carries a high risk of complications with an average reoperation rate of 22%. Varus posteromedial rotational instability fracture-dislocations have only recently been described as having the potential to cause severe long-term problems. Cadaveric studies have reinforced the need to obtain post-reduction CT scans as the size of the coronoid fragment influences the long-term stability of the elbow. Anterior dislocation with olecranon fracture has the same treatment aims as other complex dislocations with the added need to restore the extensor mechanism.

**Conclusion:**

Complex elbow dislocations are injuries with significant risk of long-term disability. There are several case-series in the literature but few studies with sufficient patient numbers to provide evidence over level IV.

## 1 INTRODUCTION

The elbow is the second most commonly dislocated joint in adults. Between 5 and 20% are associated with a fracture [1, 2] and this combination may complicate recovery. Therefore the presence of any concomitant fracture to the radius, ulna or humerus around the ipsilateral elbow joint is termed a complex dislocation [1]. Complex elbow dislocations encompass a large range of injuries from dislocations associated with undisplaced radial head fractures to significant bony injuries causing chronic instability. The aims of treatment are to achieve a stable, functional joint to allow early comfortable range of motion, and to minimise long-term sequelae. Most activities of daily living can be performed with a flexion/ extension arc of 30-130 degrees, 50 degrees of pronation and 50 degrees of supination [3]. To achieve this, it is important to understand the related anatomy, mechanisms of injury and available operative techniques for these injuries.

## 2 ANATOMY

Elbow stability is described in static and dynamic states. When the elbow is at rest, the anatomical structures providing stability are the ulnohumeral joint, medial collateral ligament complex (MCL), Lateral Collateral Ligament Complex (LCL) and the capsule [4]. These can be tested with passive stress during an examination under anaesthesia. Dynamic stability encompasses the congruity of the articulation when the muscles crossing the elbow joint contract to enable movement [7]. The primary static stabilisers of the elbow are the ulnohumeral articulation, the MCL and the LCL [5]. Of these, the MCL is the primary stabilizer in flexion, and its removal will cause instability in all positions except extension [6]. The radiohumeral articulation is a secondary stabiliser, providing up to 30% of lateral and anterior stability throughout the flexion arc [10, 11]. In valgus stress, the MCL is stretched and the radiohumeral joint compressed, making these the primary stabilizers. In varus stress, the LCL and capsule are stretched and the ulnohumeral joint is the primary stabilizer.

Cadaveric studies [11] have shown that elbow fracture dislocations are most likely to occur in a range between 15 degrees of extension and 30 degrees of flexion. This is where the MCL is least effective. Axial loading across the joint in these positions puts most stress on the anterior part of the ulnohumeral joint causing coronoid fracture and dislocation. The higher the amount of flexion within this arc, the larger the coronoid fragment is likely to be. Loading in flexion over 30 degrees is more likely to instead produce an olecranon fracture.

## 3 MECHANISM OF INJURY

Approximately 60% of complex dislocations are caused by a fall from standing height, but the fracture pattern is not always one of inherent fragility, and considerable force is required to sustain a complex dislocation in normal bone. This is reflected in two recent reviews of patient data, showing male patients in their fourth or fifth decade [12, 13]. The elbow fracture dislocations tend to occur in distinct patterns dependant on the mechanism of injury. Elbow extension with forearm supination and added valgus stress puts most strain on the ulnohumeral joint, radial head and MCL respectively, causing a posterolateral rotational instability pattern of fracture-dislocation which includes posterior dislocation with a radial head fracture and the terrible triad injury with an added coronoid fracture. An axial load to the elbow in extension and a varus stress will cause compression injury to the medial side of the elbow giving rise to coronoid fractures, and tension forces acting laterally causing LCL rupture [14]. With the elbow in more flexion, this pattern can also cause an olecranon fracture. The outcome from this mechanism is termed as varus posteromedial rotational instability. A direct blow to the posterior aspect of the flexed elbow can cause an anterior dislocation with olecranon fracture, as can a hyperextension injury.

## 4 DISLOCATION WITH RADIAL HEAD FRACTURE

As part of the posterolateral rotational instability pattern of injury, fractures occur as the anterior part of the radial head is driven into the capitellum during dislocation. Fractures can be total articular or partial articular. If fractures are partial articular, they involve the anteromedial facet of the radial head most often, followed by the anterolateral facet [15]. Fracture dislocations are associated with ligamentous tears and therefore, the function of the radial head as a secondary stabiliser of the elbow joint is important in these types of injuries. Where there is no other fracture and the dislocation is reduced, the treatment should focus less on the function as a stabiliser and more on the function of the radial head in forearm supination and pronation and the prevention of arthrosis [16, 17]. Treatment can be non-operative or operative and this will depend on the complexity of the injury as well as the comorbidities of the patient. Non-operative treatment comprises a period of immobilization to allow fracture healing before joint mobilization with physiotherapy. Immobilisation for more than 4 weeks has been shown to have poor outcomes [18]. Operative treatment can take the form of radial head ORIF, excision or replacement, and in the context of fracture dislocation will usually involve ligamentous repair. Radial head fractures are classified according to the Mason system [19] (Table **1**). Type I and II fractures associated with dislocation have good results when treated with closed reduction or ORIF [18], but fractures with more than 3 fragments do poorly when compared with radial head replacement [20, 21]. Better long term results are seen in displaced or comminuted type III fractures treated with radial head replacement rather than excision when there is instability as seen with fracture dislocations [21, 22]. When excision of the radial head is planned following a fracture-dislocation of the elbow, radial head prosthesis should always be available in case there is gross instability in examination after excision.

## 5 TERRIBLE TRIAD INJURIES

The “terrible triad” pattern is so named because the three combined injuries of elbow dislocation, radial head fracture and coronoid fracture significantly increase elbow instability and rate of complications [3-6]. The stabilizers of the elbow are disrupted from lateral to medial as the forearm supinates and is loaded [23]. The resulting damage to all primary elbow stabilisers and the secondary stabilisers of the capsule and radial head make this an injury that must be identified then treated appropriately to avoid long the term sequelae of chronic instability.

There is no specific classification for the terrible triad injury, but instead the radial head fractures and coronoid fractures are classified separately. The Mason classification [19] is used for radial head fractures and the Regan & Morrey system for coronoid fractures [24] (Table **2**). The terrible triad injury most often causes Regan & Morrey type I or II fractures and Mason type II or III fractures [25, 26].

Terrible triad injuries usually necessitate operative treatment but there has been some success with non-operative management in carefully selected patients with minimal displacement of the radial and coronoid fragments [27, 28]. These had a concentric reduction with stability beyond 30 degrees of extension after closed reduction. Coronoid fractures were small and well reduced, with radial head fractures small enough not to cause a block to rotation. 25% in one patient series [27] and 18% in another patient series [28] needed delayed operative intervention after nonsurgical treatment of a terrible triad injury. 36% had arthrosis on radiographs at follow up. Non-operative treatment involved mobilization of the elbow through its stable range using a hinged brace for 4-8 weeks. Prolonged immobilization in plaster for longer than 4 weeks is detrimental [18].

**Fig. (1):**
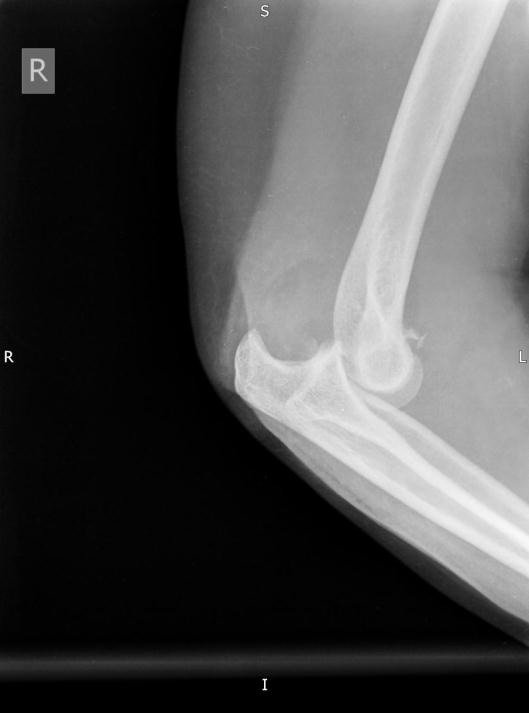
Terrible triad injuries may appear benign on plain radiographs.

**Fig. (2):**
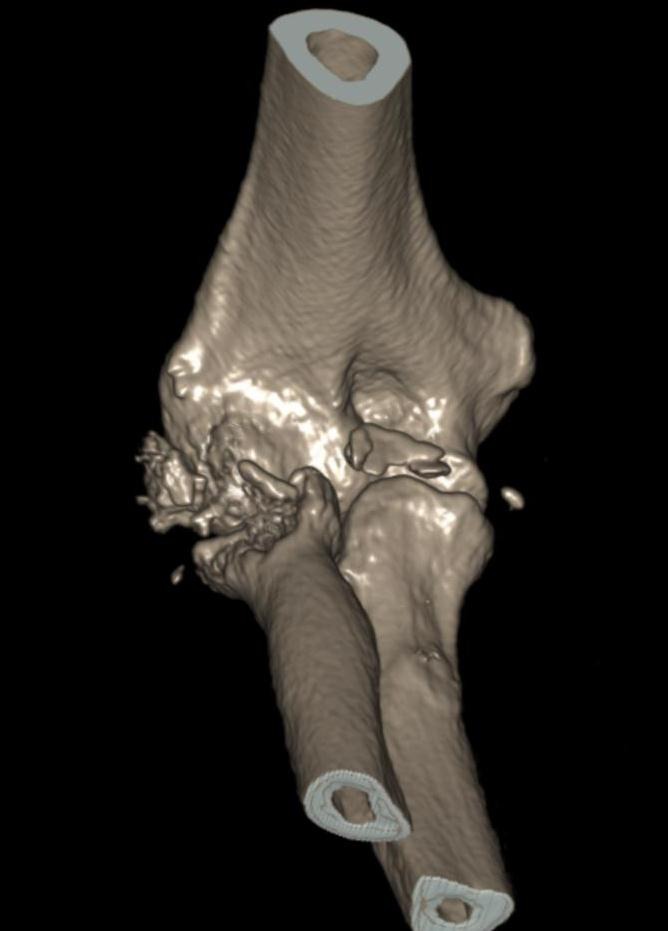
The CT scan shows a comminuted radial head fracture and coronoid fracture.

Operative treatment is often complex and should follow a thorough preoperative clinical and radiological assessment. CT scan of the elbow should be performed to assess the suitability of radial and coronoid fractures for repair or replacement [29] (Figs. **1** and **2**). Treatment within a week has been shown to be preferable to delayed or subacute operation (mean delay 7 weeks) as a better flexion arc is achieved postoperatively [30]. Traditionally, all damaged structures are repaired, including repair of the LCL to the lateral epicondyle, radiohumeral joint ORIF or replacement, coronoid process repair, MCL reconstruction [32] and capsular repair [34]. Recent studies [31, 33] have shown that reconstruction of the MCL is unnecessary in patients with a stable joint after repair of other structures, and that coronoid fractures do not need to be fixed if there is stability in the range of motion after repair of lateral structures.

Repair can be carried out through a single lateral incision if the radial head is to be replaced, as the coronoid can be accessed through the defect left by the excised radial head. A double incision technique is used if the radial head is to be fixed or if the surgeon tests stability after lateral reconstruction when deciding if to proceed to medial and coronoid repair. Usual technique is shown in Fig. (**3**). The coronoid can be repaired using sutures, anchors or screws depending on the size of the fragment and exposure. The fixation should be solid enough to allow an early range of movement without instability, and does not need to be anatomically reduced. The “suture lasso” technique has been shown to have a lower rate of malunion and a higher stability than screw fixation or using suture anchors [35].

**Fig. (4):**
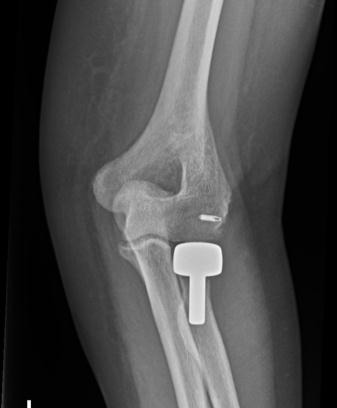
Anteroposterior and lateral radiographs after radial head replacement and lateral collateral ligament repair.

**Fig. (5):**
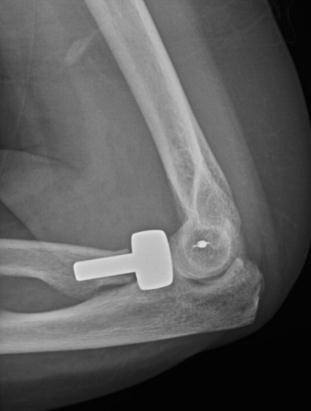
Anteroposterior and lateral radiographs after radial head replacement and lateral collateral ligament repair.

Following radial head replacement or ORIF the LCL should be repaired to the lateral condyle using suture anchors or transosseous sutures (Figs. **4** and **5**). If the elbow is unstable after repair of the coronoid, radiohumeral joint and LCL then the MCL can be repaired. Some surgeons advocate prophylactic decompression of the ulnar nerve during this stage [36]. The use of a hinged external fixator in persistent instability has been shown to improve outcomes [37, 38]. This is usually left on for 4 weeks with extension limited to 30 degrees. The flexion arc can be increased on a weekly basis, and this can be continued in a hinged brace on removal of the external fixator. If a static external fixator is used it should be limited to less than three weeks because of the propensity of the joint to become stiff.

Surgical treatment of terrible triad injuries carries a high complication rate with an average of 22% reoperation rate [39]. Complications include redislocation [6] or instability due to LCL rerupture [40], arthrosis [6, 36], non-union of radial head ORIF [41], ulnar neuropathy [30, 31, 42], radial neuropraxia [43] and heterotopic ossification [44]. The presence of arthrosis after operation for terrible triad injuries was 11.2% [39] in comparison to 36% in a separate study of non-operative treatment [28].

## 6 VARUS POSTEROMEDIAL ROTATIONAL INSTABILITY FRACTURE- DISLOCATIONS

These injuries are sustained with varus force to the elbow, causing LCL rupture and compression medially. Medial compression with varus stress characteristically produces a fracture of the anteromedial facet of the coronoid process (AMCF) [26]. Loss of these stabilisers causes subluxation or dislocation and arthrosis if left untreated [45]. This injury pattern has only recently been recognised as having the propensity to cause long term instability and arthrosis [45], and approaches to treating the injury are developing. Anteromedial facet factures can be classified according to the O’Driscoll System (Table **3**). Cadaveric studies have shown that the size of the anteromedial coronoid fragment is significant in predicting long term stability [46].

Subtype I fractures were almost as stable as a normal elbow in varus/ valgus stress and flexion/ extension when the LCL was repaired. Subtype II fractures less than 2.5mm in size were stable in flexion/ extension but unstable in varus position. The instability worsened with a larger 5mm fragment, with instability in varus and valgus positions. Subtype III fractures are unstable in varus stress and flexion/ extension even when the LCL has been repaired. These findings stress the importance of obtaining CT scans after initial reduction to assess the size and type of coronoid fracture. Non-operative treatment has been shown to be successful in patients with small minimally displaced coronoid fractures [47]. Operative treatment is necessary for larger fragments and where there is instability with smaller fragments treated non-operatively. The aim of surgery is to stabilise the joint by repairing either the LCL, coronoid fragment or both structures. There is conflicting evidence for the operative strategy; some centres advocate repair of the AMCF with LCL repair only if there is then instability on varus stress [48], and others advocate repair based on O’Driscoll classification [49]. Repair based on classification reinforces the findings from cadaveric studies [46]. Good outcomes were achieved with LCL repair only for Subgroup 1 fractures, and AMCF fixation and LCL repair for subgroup II and III Injuries. AMCF repair is usually with buttress plating and screw fixation.

## 7 ANTERIOR DISLOCATION WITH OLECRANON FRACTURE

These complex trans-olecranon fracture dislocations are relatively uncommon [50]. A direct blow to the flexed elbow can cause an anterior displacement of the ulna with resulting olecranon fracture. Hyperextension can also cause anterior dislocation, as the ulna is levered past the anterior humeral edge and the olecranon fragment pulled posteriorly by triceps as the humerus is driven through the olecranon. These are high energy injuries often caused by road traffic collisions [51, 52] and are associated with concomitant ipsilateral elbow injuries, such as segmental fracture in over 80% [53]. If an anteromedial coronoid fracture is seen with olecranon fracture and history of dislocation then the mechanism is more likely one causing varus posteromedial rotational instability, and should be treated as such with coronoid and/ or LCL repair with additional olecranon fixation. Treatment aims to restore the articular surface and extension mechanism of the elbow. Primary closed reduction should first be performed followed by CT scanning of the elbow to assess for other injuries that could lead to instability. Operative treatment is usually with tension band wiring or plate fixation followed by early mobilisation. The tension band principle relies on a solid buttress on the articular surface of the olecranon, and is not suitable in severe comminution. When used it should be followed by early mobilisation which is most effective at 30-120 degrees of active extension [54]. In severely comminuted fractures bone grafting may be necessary [55, 56]. Even in severely comminuted injuries, the primary aim of surgery should remain to restore the trochlear notch of the olecranon, as good outcomes can still be achieved [57, 59]. If reconstruction of the olecranon is not possible because of severe comminution, osteoporotic bone, concomitant injuries or patient factors then delayed fragment excision and triceps advancement may be used to salvage the extensor mechanism [59]. Frequent complications include arthrosis in up to 70% and ulnar nerve dysfunction in up to 25% of patients [60].

## 8 OTHER COMPLEX DISLOCATIONS

Other patterns of injury may exist and case reports reflect the multitude of ways in which forces can disrupt the elbow joint. Posterior dislocation with olecranon fracture has been described with [61] and without coronoid fracture [62]. There have also been reported terrible triad fractures with triceps avulsion [65]. These injuries were associated with high energy mechanisms. A variant of the posterior dislocation has been described with biepicondylar humeral fracture in a seventeen year old male patient [63], and also with radial shaft fracture and radial head dislocation [64]. This highlights the need of the surgeon to thoroughly assess the patient with routine examination of structures around the elbow that are not usually associated with complex dislocation. The importance of examining the joint below and above that affected by injury is highlighted in a case report of a young man with dislocation of the elbow and a missed fracture dislocation of the carpus (floating forearm) [66]. These cases are uncommon, but remind us that elbow fracture dislocations and terrible triad injuries are often high-energy, and patients often have concomitant fractures or soft tissue injuries in the ipsilateral limb that may delay recovery if missed. Treatment in all these case reports is based on the aim of reconstruction of joint congruency and stabilising structures.

## 9 DISCUSSION

The pathoanatomy and mechanism of complex dislocations and terrible triad injuries is well understood due to investigations with cadaveric studies [11, 46]. Subsequently the structures acting as primary (ulnohumeral joint, MCL, LCL) and secondary (radiohumeral joint, capsule, muscles and tendons crossing the joint) stabilisers have been better understood. Although there is no formal classification system for complex dislocations, these studies have led to a broad division based on the mechanism of injury and direction of instability. There is consensus opinion in the literature that elbow complex dislocations should be managed by immediate primary closed reduction followed by a thorough clinical examination and CT scan of the elbow. There is also consensus opinion that dislocations with radial head fracture should be treated with radial head replacement in Mason III injuries or Mason II fractures where the joint cannot be reconstructed [20-22]. Terrible triad injuries were initially treated with operative repair of all the damaged structures; LCL, Radial head, coronoid and MCL. There seems to be a trend in the literature towards repairing lateral structures only, applying stress to the joint then only repairing medial structures if there is instability [31-34]. The term “terrible triad” was only coined in 1996, and the long term outcomes of its more conservative operative treatment has not been shown in large series. As a relatively uncommon injury there are no randomised controlled trials in the literature to assess the best surgical algorithm. Whichever algorithm is followed the aim of surgery is the same: to achieve an outcome where the joint is stable and has a pain free functional range of motion. The importance of AMCF and LCL injuries in causing long term instability and arthrosis has only been recognised in the last decade. Several studies [45, 46, 48, 49] have investigated this pattern of fracture-dislocation and describe effective operative management. Evidence for coronoid fixation seems robust, but the absence of studies with large numbers of patients means the optimal operative treatment for anteromedial coronoid rim fractures is still in doubt, with the largest study only providing level III evidence. Treatment of olecranon fracture dislocations is well established. After closed reduction of the dislocation the treatment is the same as for a simple olecranon fracture; restoration of the articular surface with internal fixation and early mobilisation [56, 57, 60].

## CONCLUSION

Complex elbow dislocations are injuries with significant risk of long-term disability, which may be made worse with inappropriate treatment due to a missed diagnosis. It is essential to perform a thorough examination of the patient bearing in mind that complex dislocations are frequently high-energy injuries. CT scanning as an adjunct to diagnosis is invaluable as the true nature of radial head and coronoid fractures may not be apparent on plain radiographs. There is no unifying classification system for complex dislocations or terrible triad injury. The creation of the trauma network in the UK has concentrated subspecialists geographically and this will lead to more opportunities for collaborative research in these relatively uncommon but severe injuries.

## Figures and Tables

**Fig. (3):**
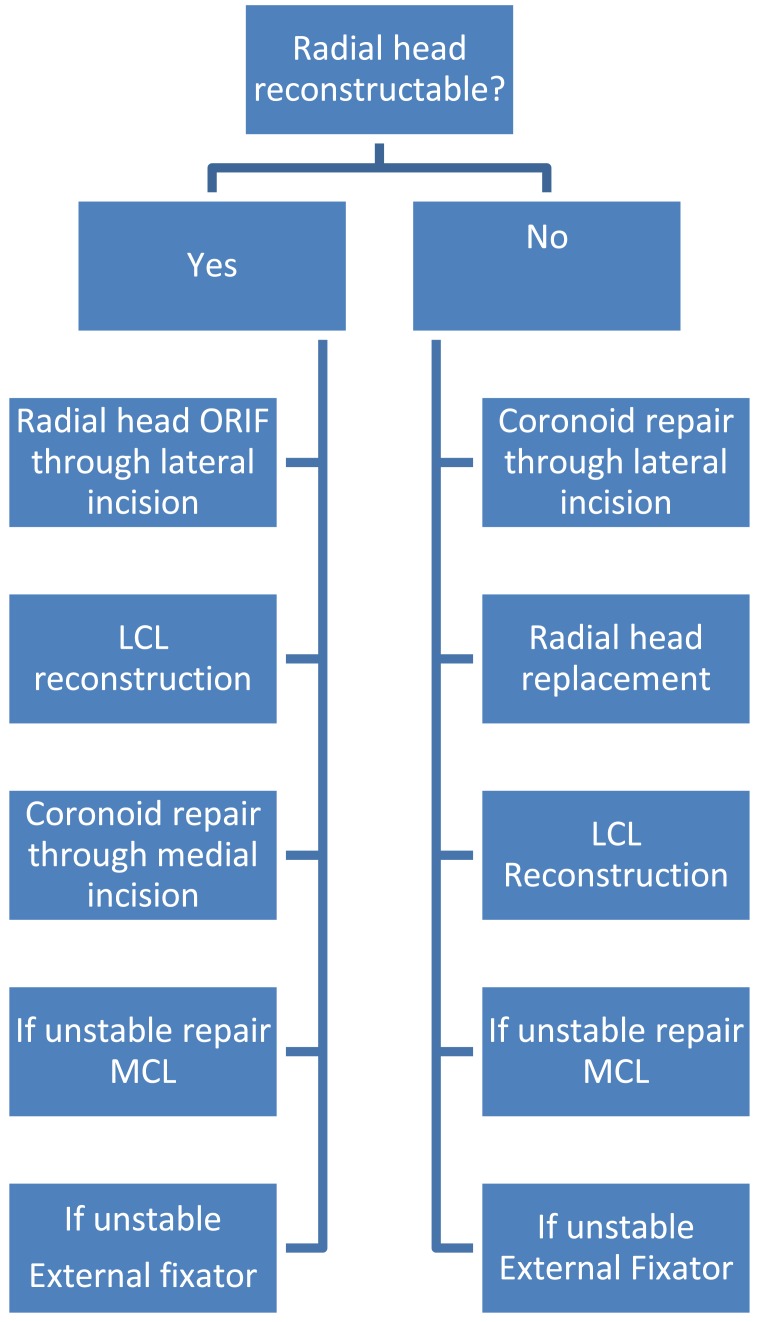
Algorithm for surgical treatment of terrible triad elbow injuries.

**Table 1:** The Mason system for classification of radial head fractures.

Mason Type	Injury Pattern
**I**	Undisplaced
**II**	Displaced Partial Articular
**III**	Multifragmentory/ Displaced Total Articular

**Table 2:** The Regan & Morrey system for classification of coronoid fractures.

Type	Injury Pattern
**I**	Coronoid tip fractures
**II**	Fracture of less than 50% of height of coronoid
**III**	Fracture of more than 50% of height of coronoid

**Table 3:** O’Driscoll Classification of anteromedial coronoid fractures.

**Subgroup**	–
**I**	Fracture of the Anteromedial Rim
**II**	Fracture of the Anteromedial rim and tip
**III**	Fracture of the Anteromedial rim and sublime tubercle
